# Optimization of the antibiotic management of diabetic foot infections: protocol for two randomized controlled trials

**DOI:** 10.1186/s13063-019-4006-z

**Published:** 2020-01-08

**Authors:** Felix Waibel, Martin Berli, Sabrina Catanzaro, Kati Sairanen, Madlaina Schöni, Thomas Böni, Jan Burkhard, Dominique Holy, Tanja Huber, Maik Bertram, Karin Läubli, Dario Frustaci, Andrea Rosskopf, Sander Botter, Ilker Uçkay

**Affiliations:** 1 Team Technical Orthopedics, Department of Orthopedic Surgery, Balgrist University Hospital, Zurich, Switzerland; 2 Unit for Clinical and Applied Research, Balgrist University Hospital, Zurich, Switzerland; 3 Internal Medicine, Balgrist University Hospital, Zurich, Switzerland; 4 Hospital Pharmacy, Balgrist University Hospital, Zurich, Switzerland; 5 Nursing Department, Balgrist University Hospital, Zurich, Switzerland; 6 Physiotherapy, Balgrist University Hospital, Zurich, Switzerland; 7 BioBanking, Balgrist Campus AG, Balgrist University Hospital, Zurich, Switzerland; 8 Radiology, Balgrist University Hospital, Zurich, Switzerland; 9 Infectiology, Balgrist University Hospital, Forchstrasse 340, 8008 Zurich, Switzerland

**Keywords:** Diabetic foot infections, Osteomyelitis, Partial amputation, Antibiotic duration, Remission, Adverse events

## Abstract

**Background:**

Few studies have addressed the appropriate duration of antibiotic therapy for diabetic foot infections (DFI) with or without amputation. We will perform two randomized clinical trials (RCTs) to reduce the antibiotic use and associated adverse events in DFI.

**Methods:**

We hypothesize that shorter durations of postdebridement systemic antibiotic therapy are noninferior (10% margin, 80% power, alpha 5%) to existing (long) durations and we will perform two unblinded RCTs with a total of 400 DFI episodes (randomization 1:1) from 2019 to 2022. The primary outcome for both RCTs is remission of infection after a minimal follow-up of 2 months. The secondary outcomes for both RCTs are the incidence of adverse events and the overall treatment costs. The first RCT will allocate the total therapeutic amputations in two arms of 50 patients each: 1 versus 3 weeks of antibiotic therapy for residual osteomyelitis (positive microbiological samples of the residual bone stump); or 1 versus 4 days for remaining soft tissue infection. The second RCT will randomize the conservative approach (only surgical debridement without *in toto* amputation) in two arms with 50 patients each: 10 versus 20 days of antibiotic therapy for soft tissue infections; and 3 versus 6 weeks for osteomyelitis. All participants will have professional wound debridement, adequate off-loading, angiology evaluation, and a concomitant surgical, re-educational, podiatric, internist and infectiology care. During the surgeries, we will collect tissues for BioBanking and future laboratory studies.

**Discussion:**

Both parallel RCTs will respond to frequent questions regarding the duration of antibiotic use in the both major subsets of DFIs, to ensure the quality of care, and to avoid unnecessary excesses in terms of surgery and antibiotic use.

**Trial registration:**

ClinicalTrials.gov, NCT04081792. Registered on 4 September 2019.

## Introduction

### Background and rationale

Diabetic foot infections (DFIs) are frequent and harbor a high burden of morbidity, costs, and recurrences worldwide [1]. Knowing the potential for poor outcomes, many clinicians tend to treat DFIs with prolonged antibiotic therapy, with concomitant side effects, spreading of antibiotic resistance, and increasing associated costs [1, 2]. In contrast, scientific data from the few comparative trials available have shown that 1–2 weeks of antibiotic treatment is sufficient for most diabetic foot soft tissue infections, and 4–6 weeks appears adequate for (unresected) infected bone [1–3]. A randomized trial compared a 6-week against a 12-week course of antibiotic therapy, without concomitant surgery, for diabetic foot osteomyelitis (DFO) and found similar outcomes. This study set the maximal duration at 6 weeks for the conservative treatment of DFO, but shorter durations have not been evaluated [4]. A pilot study in Geneva is still recruiting, and randomizes postsurgical antibiotic therapy between 10 and 20 days for soft tissue DFI and between 3 and 6 weeks for DFO, and has found no difference in terms of remission in two interim analyses (ClinicalTrials.gov NCT03615807) [5]. Another recent case–control study with 1018 DFI episodes equally failed to determine the optimal duration of systemic antibiotic therapy in all substrata of DFIs, but advocated that current therapy schema might be too long [6]. Clearly, there is room for improving antibiotic stewardship efforts in DFI [3] and interest for randomized controlled trials (RCTs) on DFI.

## Methods

### Study setting

The Balgrist University Hospital in Zurich is a tertiary referral center for DFI and amputations (emergency and elective consultations with a 24-h service) and is affiliated to the University of Zurich. The center has a multidisciplinary team composed of four surgeons for DFI, three internist physicians, a hospital pharmacist, five specialized wound nurses, two Foot Care nurses, musculoskeletal expert radiologists, a diabetes nurse, three nutritionists, a shoemaker, a prosthesis specialist, and up to four infectious diseases physicians who are specialized in orthopedic infections. Moreover, this team is supported by an in-house company providing orthopedic footwear (Balgrist Tec) and individual adaptations of off-loading devices, a re-education unit, physical therapy, a research campus (Balgrist Campus) with a BioBank, and a Unit for Clinical and Applied Research with nine study nurses and three personnel with experience in biostatistics and investigative designs (www.balgrist.ch). This research unit runs a register for DFIs and DFOs. This register is presumably the largest in Switzerland. Our potential of recruitment oscillates between one and four new DFI episodes (hospitalized patients and outpatients) per week. This study will start at the Balgrist, but it is expandable to other national or international centers.

### Study: two concomitant prospective-randomized trials

We will conduct two concomitant, prospective, RCTs on the duration of postsurgical systemic antibiotic therapies for DFIs, including DFOs. The first RCT (on residual infection after amputation) will have the primary study question of whether systemic antibiotic therapy can be shortened in amputated patients with eventual residual soft tissue infection or residual stump osteitis. The secondary study outcomes are the incidence of adverse events and overall costs related to the treatment. The second RCT (on the duration of systemic antibiotic therapy in nonresected infections) will have the primary study question of whether antibiotic therapy can be shortened in nonamputated patients with soft tissue infections and osteitis. The secondary study outcomes are the incidence of adverse events and overall costs related to the treatment.

### Definitions and eligibility criteria for participants

We will class DFI episodes according to the severity of infections and Infectious Diseases Society of America criteria [2]. Mild infection is defined as having ≥2 manifestations of local inflammation (swelling or induration, erythema, tenderness, warmth, purulent discharge). Moderate DFI is erythema >2 cm, or involving structures deeper than the subcutaneous tissues [2]. We define DFO as a bone infection with any positive microbiological, histological and/or radiological evidence of bone involvement. We define remission as the absence of clinical, anamnestic, radiologic and/or laboratory signs of former infection. Of note, new or persistent necrosis, fracture, Charcot deformity or ulceration can be interpreted as remission as long there are no signs of infection. The anatomical area defining DFI for the study terminates at the ankle joints, but participants are eligible with leg infections as long as these originate in their diabetic foot. Table 1 lists the inclusion and exclusion criteria.

**Table 1:** Inclusion and exclusion criteria for both randomized clinical trials

	First randomized trial (attempted therapeutic amputation for infection)	Second randomized trial (only debridement for infection; conservative treatment)
Inclusion criteria	• Diabetic foot infection	• Diabetic foot infection
	• Age ≥18 years	• Age ≥18 years
	• At least 2 months of follow-up	• At least 2 months of follow-up
	• Acceptance of local wound care, off-loading and revascularization (if clinically necessary)	• Acceptance of local wound care, off-loading and revascularization (if clinically necessary) • Osteomyelitis limited to bone contact and cortices in x-ray
Exclusion criteria	• >5 cm distance between amputation level and infection	• Therapeutic amputation
	• Any concomitant infection requiring more than 5 days of systemic antibiotic therapy • Osteosynthesis material not removed (if any)	• Any concomitant infection requiring more than 10 days of systemic antibiotic therapy • Has received >96 h of potentially effective systemic antibiotic therapy and the wounds been clinically improving • Destructive osteomyelitis with fractures, sequestra, shattering upon contact, vanishing beyond cortical involvement • Material-related infection

### Interventions and study conduct

For both RCTs we maintain current therapeutic practices. Basically, amputation or disarticulation is foreseen for DFO with advanced bone destruction and terminal (painful) ischemia, but not for DFI per se. The amputation level will be kept as distal and as minimal as possible. We will perform amputation at a level determined by magnetic resonance imaging (MRI) and mechanical properties. All surgeries will be performed with the participation of an experienced surgeon. The patient will be invited to participate and will be allocated to a short or a long antibiotic treatment arm; further allocation depends of the surgical indications (amputation or conservative approach). The inclusion can occur until day 4 of surgery or effective antibiotic therapy.

#### Surgical indication: amputation

If the clinicians and the patient decide to proceed with amputation, the patient participate in the first RCT. If there is residual postamputation infection remaining either in the soft tissue or the bone, the patient will be randomized to 1 versus 4 days of antibiotic therapy for residual soft tissue infection or 1 versus 3 weeks of therapy for an eventual residual proximal stump osteitis.

All antibiotics will be stopped if no bacterial growth is seen at day 4, or according to the randomization arm.

#### Surgical indication: debridement without amputation

If the clinicians and the patient decide for debridement only (no therapeutic amputation), then the patient participates in the second RCT and will be randomized to 10 versus 20 days of antibiotic therapy for soft tissue infections, or 3 versus 6 weeks of therapy for nonamputated osteomyelitis.

**Fig. 1:**
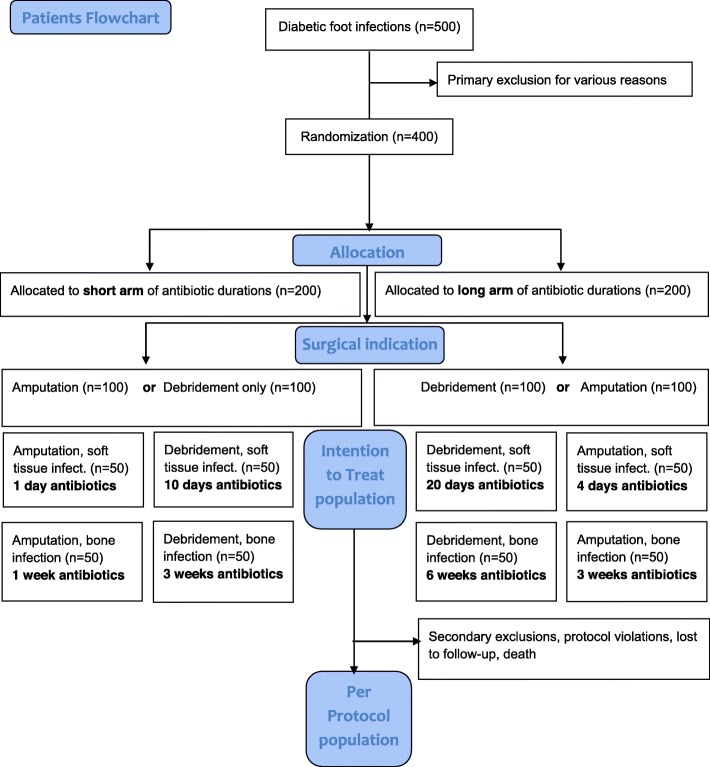
Study flowchart; Consort flow diagram

If the patient cannot participate in one RCT, they can participate in the other (Fig. 1).

Table 2 shows the variables of interest in each of the two RCTs. The follow-up will be active (regular clinical consultations by the study investigators) until 2 months postoperatively, and passive (notification of recurrence; e.g., telephone contact) at 12 months postoperatively.

**Table 2:** List of prospectively assessed variables during both randomized trials

− Patient’s general descriptive characteristics: birth date, age, sex, hospitalization number, pertinent actual and past comorbidities, current medication, ischemia, coronary heart disease, depression, stroke, heart insufficiency, duration of diabetes, glycated hemoglobin, insulin therapy, creatinine clearance, dialysis, hypertonia, statin use, anticoagulation, smoking habits, alcohol intake, American Society of Anesthesiologists (ASA) score, Frailty Score according to Fried, patient nutritional status	
− General diabetic foot problems: type and duration of presurgical antibiotic therapy, presurgical hospitalizations, presurgical microbiological results including antibiotic susceptibility profiles, presurgical podiatric care, anatomical localization of infection, type and side of foot problem, past amputations, past foot surgery, presence and type of osteosynthesis material in the foot, PEDIS Score, Wound Score and localization, Charcot foot, transcutaneous oxygen tensions, peripheral arterial disease staging, ankle-brachial index, angioplasty, x-rays, magnetic resonance imaging and other radiological results of the foot, number and type of surgeries for the actual problem	
− Diabetic foot infection: presence of soft tissue infection, osteomyelitis, bacteremia, iterative serum C-reactive peptide levels, fever, pathogens and antibiotic susceptibility profiles	
− Treatment variables: number and type of surgeries, amputation techniques, type of dressings, number and types of intraoperative samples, and duration, type, numbers and administration route of all antimicrobials, infectiology consultations, other medical, physiotherapeutic, ergotherapeutic, and nursing consultations and notes	
− Administrative data: total costs, length of hospital stay, length of re-education, number of ambulatory consultations, first and last consultation date, follow-up duration, BioBanking data	
− Outcome parameters: remission, clinical and microbiological recurrences, progressive ischemia, adverse events, patient satisfaction per questionnaire at 2 months after end of treatment, eventual prostheses, and type of off-loading devices, rehospitalization and retreatment elsewhere, Frailty Score according to Fried, total treatment costs, and the nutritional status at Test-of-Cure-visit	

### BioBanking

If the patient undergoes surgery we will ask to sample the intraoperative tissue for BioBanking for eventual further research, or completeness of the current studies. The BioBank will store intraoperative specimens at ambient temperature (15–25 °C) in the Balgrist Campus. The storage will be anonymized for 10 years and financed by external grants.

### Magnetic resonance imaging

At Balgrist University Hospitals, each patient suspected as having DFO has conventional x-rays and MRI examinations as part of our standard clinical protocol. For this study, no patient will have scintigraphy in addition to the MRI. The standard MRI examination will be performed before surgery as part of the usual clinical approach. We will not test any new software. Neither RCT will demand additional radiologic examinations only for study reasons.

### Prior antibiotic therapy

A microbiologically effective antibiotic therapy beyond 96 h prior to screening is an exclusion criterion. In contrast, we will allow a 72-h window before debridement, independently of the duration of prior antibiotic administration. However, if the patient requires a new antibiotic agent based on microbiological results, independently of the duration of prior ineffective antibiotic therapy, there will be no minimal window or maximal predebridement antibiotic duration and the patient can be included into both RCTs.

The antibiotic therapy is administered according to the Infectious Diseases Society of America guidelines [2]. Initially, it is either empiric or targeted to the results of preoperative information. After 2–4 days, it becomes targeted to the susceptibility profile. The choice of the agent, and its administration route (oral or parenteral), is at the discretion of the treating clinicians. Nonetheless, for both RCTs, and to achieve a minimal homogeneity, we establish a list of “allowed antibiotics” (Table 3). We will avoid placebos, topical antibiotics and antiseptics, except for the eventual preincisional skin disinfection. Likewise, anesthesiologists will remain free to administer routine perioperative prophylaxis (cefuroxime, vancomycin, or clindamycin for up to three doses) if they judge it to be indicated. Finally, we will collect the packages from the prescriptions during the outpatient treatment as a surrogate of proof of the patient’s antibiotic intake.

**Table 3:** List of allowed antibiotic treatments (empirical or targeted)

Antibiotic agent	Allowed dosing regimens	Allowed daily total range
Levofloxacin PO	750 mg every 24 h or 500 mg every 12 h	750–1000 mg
Ciprofloxacin PO	750 mg every 24 h or 500 mg every 12 h	750–1000 mg
Amoxicillin/clavulanate PO	500/125 mg every 12 h or every 8 h	1000/250 mg to 1500/375 mg
Amoxicillin/clavulanate IV	1000/200 mg every 12 h or every 8 h	2000/400 mg to 3000/600 mg
Cefuroxime IV	1500 mg every 8 h	4500 mg
Ceftriaxone IV	2000 mg every 24 h	2000 mg
Co-trimoxazole PO	960 mg every 12 h or every 8 h	1920–2880 mg
Clindamycin PO	300 mg or 450 mg every 6 h	1200–1800 mg
Doxycycline PO	100 mg every 12 h	200 mg
Linezolid PO	600 mg every 12 h	1200 mg
Linezolid IV	600 mg every 12 h	1200 mg
Metronidazole PO	500 mg every 8 h or 500 mg every 6 h	1200–2000 mg
Metronidazole IV	500 mg every 8 h or every 6 h	1500–2000 mg
Vancomycin IV	15 mg/kg every 12 h	According to serum through levels, 10–20 mg/L
Meropenem IV	1 g or 2 g every 12 h or every 8 h	2–6 g
Piperacillin/tazobactam IV	4000/500 mg every 8 h	1200/1500 mg (12 g/1.5 g)

*IV* intravenous therapy, *PO* oral therapy

#### Pregnancy and breast-feeding women

In these studies, the antibiotics and surgeries have no specific relationship with pregnancy or breast-feeding women and their children. Additionally, the study population is likely not to reveal women at procreating age. Formally, we will not exclude pregnant and breast-feeding women, but the investigators will avoid antibiotics that are considered possibly detrimental for pregnant or breast-feeding women according to the Swiss Compendium (www.compendium.ch).

### Risks for the study participants

Besides the retrospective identification of patients in both RCTs, we ignore particular risks. For BioBanking specifically, a theoretical risk could be the detection of unknown pathologies. In such case, the investigators will engage to inform the patient orally or by letter, if they did not refuse it previously. Concerning both RCTs, a theoretical risk could be a higher incidence of recurrences in the corresponding short antibiotic arms.

### Diabetic ulcer care and pressure relief

Standard diabetic ulcer foot care will include wound debridement (during hospitalization and visits and only if clinically indicated), daily care with dressing changes, pressure off-loading and professional diabetes control. Off-loading is defined as avoidance of all mechanical stress on the injured extremity. Because off-loading is so critical to the healing process, we will instruct patients to wear the device at all times except when bathing and to use a device at all times when walking or standing is required, and eventually also during night rest. Strategies for off-loading will be standardized as follows. All ulcers on the bottom of the foot will be fitted with an off-loading device during the baseline visit 1. The size of the off-loading device (walker) will be determined based on the patient’s correct shoe size. We will insert the appropriate size of insole into the device. Once the target ulcer has been debrided, cleansed, dressed and secured, we will apply the device according to the manufacturer’s instructions for use.

### Randomization and allocation procedures

The unblinded allocation to a short or a long antibiotic duration arm in both RCTs will occur electronically in a 1:1 ratio (randomization without blocked or matched variables). The result will be dichotomous. It will be either the “short arm”, or the “long arm” of antibiotic therapy. In a further step, the surgical indication (amputation versus conservative therapy with debridement), as well as the infection site (soft tissue versus osteitis) will finally determine the exact study arm and the corresponding antibiotic duration. We will use freely available randomization programs (e.g., www.randomizer.org). The Principal Investigator, the Sponsor and two dedicated study nurses only will be allowed to randomize and to implement. They will conceal the randomization procedure electronically, and as printouts in the study documents.

### Monitoring

The Unit for Clinical and Applied Research will assign an independent monitor (with experience in prospective RCTs) to the study. All patient files, notes and copies of laboratory and medical test results must be available for monitoring. The monitor will verify all, or a part of, the case report forms (CRF), data and written informed consents. One monitoring visit at the investigator’s site prior to the start and twice during the study will be organized by the Sponsor. Furthermore, there will be a close-out visit at the study end.

#### Audits and inspections

A quality assurance audit/inspection of this study may be conducted by the competent authorities. The quality assurance auditor/inspector will have access to all medical records, the investigator’s study-related files and correspondence, and the informed consent documentation. The investigator will allow the persons responsible for the audit or the inspection to have access to the source data/documents and to answer any questions arising.

### Timetables and study visits

**Fig. 2:**
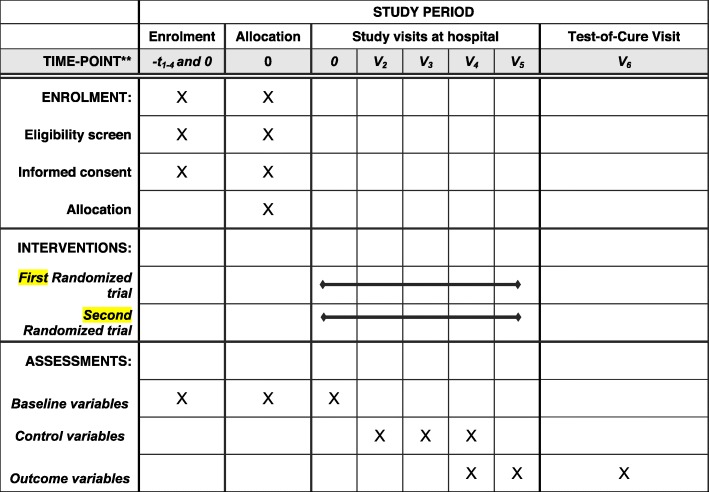
SPIRIT flowchart of time events of both randomized clinical trials in this study. IV intravenous

For both RCTs, we need 36 months starting in September 2019. Table 4 displays the overall study timeline. The SPIRIT figure (Fig. 2) shows the time points for the study visits. All study participants will have weekly assessments, an end-of-treatment visit and a test-of-cure visit 2 months after that. Another visit will take place at 12 months which is only interested in the question if there has been a recurrence during the year following treatment. This last piece of information can be gathered via a telephone call, a patient visit, or by the general practitioner of the patient, or from the hospital’s medical files. During the active study period, the assessments will be identical for both RCTs regarding the study objectives (primary and secondary outcomes) and their individual study arms (soft tissue versus bone infection), with the only exception that patients in the shorter antibiotic arms will terminate the study earlier by 1 to 3 weeks. The aggregation of study-related information, laboratory data and clinical assessments at each study visit time point is summarized in Table 5.

**Table 4:**
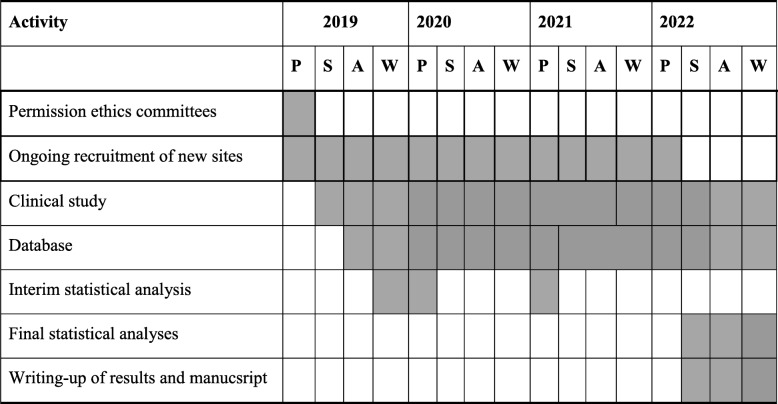
Timetable of the study

*A* autumn, *P* spring, *S* summer, *W* winter

Shaded cells = Study-related activities by calendar periods

**Table 5:** Assessments during the study visits in the randomized clinical trials (RCTs)

First RCT (attempted therapeutic amputation)*						
Study visits	Baseline visit 1	Visit 2	Visit 3	Visit 4; end of trial (EOT)	Visit 5; test of cure	
Time points	Day 0–1	Day 8 (±2 days)	Day 15 (±2 days)	Day 21 (±2 days)	20–30 days after EOT	
Identity; MRI examination	x					
Inclusion/exclusion criteria	x		x			
Informed consent	x					
Demographics	x					
Medical history	x				x	
Clinical assessment of infection	x	x	x	x	x	
Intraoperative sampling	x					
Control of compliance		x	x	x	x	
Adverse events		x	x		x	
Study end (control)					x	
Second RCT (infection only debrided, conservative treatment)*						
Study visits	Baseline visit 1	Visit 2	Visit 3	Visit 4	Visit 5; end of trial (EOT)	Visit 6; test of cure
Time points	Day 0–1	Day 8 (±2 days)	Day 15 (±2 days)	Day 21 (±2 days)	Day 40 (±2 days)	20–30 days after EOT
Identity; MRI examination	x	x	x	x	x	x
Inclusion/exclusion criteria	x		x			
Informed consent	x					
Demographics	x					
Medical history	x					x
Clinical assessment of infection	x	x	x	x	x	x
Intraoperative sampling	x					
Control of compliance		x	x	x	x	x
Adverse events		x	x	x	x	x
Study end (control)						x

*In both RCTs, we will use clinically sampled data, laboratory and radiology results. There will be no special sampling purely for the RCT. A second and final control will happen at 12 months after treatment

*MRI* magnetic resonance imaging

### Statistical analyses, study objectives and sample sizes

#### Statistical approach to the study objectives and statistical analysis plan

The primary objective for both RCTs is the remission of infection at 2 months postoperative follow-up. The contrast to remission is recurrence. We will classify recurrence as “clinical recurrence” with recurrent or new infection in the former infection site, and as “microbiological recurrence” with the same pathogen(s) as for the index infection at the same infection localization. The secondary objectives are identical for both RCTs: the risk for adverse events in each randomization arm and the overall treatment costs.

#### Statistical techniques, study design and sample size calculations

Statistically speaking, both RCTs are simple to analyze and simple in their design. Therefore, we will not have a formal and separate statistical analysis plan. The Sponsor and the Principal Investigator wrote the analytic strategy together. Both RCTs are exactly the same noninferiority studies, without adaptive study designs. Moreover, we apply the same noninferiority design for the primary outcome of remission, as well as for the secondary outcome of adverse events. Regarding the secondary outcome of treatment costs we do not plan any noninferiority requirements, since DFIs are multifaceted diseases with substantial interference with other expensive pathologies. The study objectives clinical remission and adverse events will be binomial variables; the objective overall costs outcomes will be expressed as continuous variables.

The expected clinical remission is set at 80% for each study arm in both RCTs. Noninferior margins are set at 20%, with power 80% and alpha 5%. Excluding some anticipated drop-outs, we require 2 × 50 episodes regarding soft tissue infections, and 2 × 50 episodes for residual DFO for the first RCT (residual infection after amputation). For the second RCT (duration of antibiotic therapy in nonresected DFI), we equally require 2 × 50 episodes for soft tissue infections and 2 × 50 cases for DFO (Fig. 2). Hence, the total overall study population will be 400 participants, while an individual patient can participate several times in either RCT provided that each DFI episode occurs at another infection site.

For both RCTs, a Data Monitoring Committee will perform interim analysis after the inclusion of the first 40 episodes and again at 100 episodes, and will decide on the continuation of the studies. If there are overt differences in terms of remission between the short and long antibiotic arms in all subsets of DFIs, we will terminate the study. During these interim analyses, we will equally check if the expected statistical power for the final analysis will not fall under our arbitrary limits of unacceptability. If this power becomes lower than 50%, we will consider the trial no longer ethical. To balance a potential loss of power, we also may recruit 50 supplementary participants per RCT if the trial has not been stopped.

#### Methods of data aggregation

The analyses will be based on descriptive statistics (numbers, median values with ranges) and group comparisons (Pearson χ^2^ test or the Fisher exact test for categorical variables; the Wilcoxon rank-sum test for (nonparametric) continuous variables). Multivariate, unmatched, cluster-controlled (at a patient level) Cox regression analyses will adjust for the large case mix that we expect. If the study becomes multicentric, another cluster level will be allocated to the individual hospitals. The Cox regression is the only survival analysis we will perform. We will not use time series analyses, log rank tests or Kaplan-Meyer curves because of the substantial case mix and the limited determination of the clinical variable “antibiotic use” among all complex and mixed pathologies associated with diabetic foot problems.

Variables with a univariate association of *P* < 0.2 will be included in the final model, while the duration of antibiotic therapy, the number of surgical debridements and the presence of angioplasties will be automatically incorporated into the final model. A minimal follow-up time of at least 2 months postsurgery is required to be included in the multivariable models. We will check for collinearity and interaction (effect modification) by interaction terms and Mantel-Haenszel estimates. The individual start in the Cox regression analysis will be the date of first debridement. The individual follow-up times will be censored at 12 months, death, or the date before the loss to follow-up. Since our RCTs will be prospective and the study population well balanced, we anticipate few missing data. Consequently, we plan no imputations and will not perform matched analyses. The requirement for the supposed noninferiority will be computed using a χ^2^ analysis with the real differences displayed as percentage points and 90% confidence intervals in both outcome assessments (remission; adverse events) for each RCT and for each study population separately. The (two-tailed) statistical significance level will be set at *P* < 0.05.

#### Presentation of the study populations

For both RCTS, we will publish the outcomes “remission” and “adverse events” as the intent-to-treat (ITT) and the per-protocol (PP) database. We will not use modified ITT populations. The ITT participants, who have signed the consent letter, will consist of all randomized DFI episodes, even if the patient drops out of the study or if there is protocol violation. The PP population will consist of all patients completing the study and who have not deviated significantly from the protocol. Importantly, the PP analysis will be restricted to the participants who fulfil the entire protocol requirements in terms of the eligibility, adherence to the intervention, and outcome assessment. It will represent the best-case scenario being studied. Both RCTs already incorporate two subgroups (soft tissue versus bone infections). This makes a total of four subgroup analyses. There are no further subgroups planned (Fig. 2). However, we reserve the right to perform further (yet unidentified) subgroup analyses if we detect by chance any substantial particularities in the final results.

### Ethical and regulatory aspects

#### Study registration

The study is registered in the Swiss Federal Complementary Database (“Portal“) and in the international registry ClinicalTrials.gov (NCT04081792). This study only will make use of antibiotics that are already authorized in Switzerland for DFO and corresponding soft tissue infections. The indication and the dosage will be used in accordance with the prescribing information and international guidelines [2]. All drugs and doses in this study will be commonly used agents and related doses. The study protocol will not change without prior Sponsor and Ethical Committee approval. Amendments will be reported. Premature interruption will be reported within 30 days. The regular end of the study will be reported to the Ethical Committee within 90 days, and the final study report shall be submitted within 1 year after study end. The Ethical Committee and authorities will receive annual safety reports and are informed about the study stop/end. The study will be carried out in accordance with the protocol and with principles in the current version of the Helsinki Declaration, Good Clinical Practice, and the Swiss regulatory requirements.

#### Patient information and informed consent

We will inform potential participants about the study, its voluntary nature, the procedures involved, the expected duration, any potential risks and benefits and any potential discomfort. All participants will be provided an information sheet and an informed consent form. The original form remains in the study records. For the BioBank, the participants will sign a general consent regarding personal clinical data and biologic material. The investigators will uphold the principle of the participant’s right to privacy and that they shall comply with applicable privacy laws. Subject confidentiality will be further ensured by using code numbers corresponding to the computer files. For data verification, the Ethics Committee and regulatory authorities may require access to relevant medical records, including the participants’ medical history.

#### Early termination of the study

The Sponsor may terminate the study prematurely in certain circumstances; for example, ethical concerns, insufficient recruitment, when the safety of the participants is at risk, alterations in accepted clinical practice making continuation unwise and early evidence of benefit or harm of the experimental intervention. All patients will be free to withdraw from participation at any time, for any reason, and without prejudice. The reason for withdrawal should be documented wherever possible. The withdrawal will not affect the actual medical assistance or future treatments. On rare occasions, the investigators may terminate a patient’s participation to protect their best interest. After study termination, the evaluations required at the next scheduled clinical visits will remain.

### Safety

During the entire study duration, all adverse events will be recorded, fully investigated and documented in source documents and CRFs. The Sponsor will submit an annual safety report to the local Ethics Committee. For both RCTs, a Data Monitoring Committee will perform interim analysis after the inclusion of the first 40 episodes, and again at 100 episodes, and will decide on the continuation of the studies. This Committee will consist of a urologist surgeon and an anesthesiologist not involved in the study or in the future author lists.

#### Treatment by specialists

All surgeries will be performed with the participation of an experienced surgeon. The antibiotic therapy will be ordered by internists and infectious diseases physicians with therapeutic and academic experience in DFI treatments. The current medications of the study patients, as well as possible interactions, will be controlled during hospitalization by the Head of Pharmacy of Balgrist University Hospital and by the internists on a weekly basis. The Infectious Diseases physicians and surgeons will ensure this drug surveillance during the outpatient periods.

#### Definition and assessment of (serious) adverse events and other safety-related events

An adverse event (AE) is any medical occurrence in a study participant which does not necessarily have a causal relationship with the study procedure. A serious AE (SAE) is classified as any untoward medical occurrence that results in death, is life-threatening, requires inpatient hospitalization or prolongation of hospitalization, or persistent or significant disability. Participants with ongoing SAEs at study termination will be followed until recovery or stabilization after termination. The investigators will make a causality assessment. All SAEs shall be reported within 24 h to the Sponsor/Investigator. SAEs resulting in death will be reported to the Ethics Committee within 7 days. Patients will adverse events who leave the study will be treated off-study, without restriction, at the study sites.

#### Data handling and record keeping/archiving

We will save data using the secured software REDCap®. When the study is terminated, the data will be saved in the same system. During the usual clinical treatment, all health care workers and administrators at Balgrist University Hospital will have access to the clinical data. After the end of therapy, however, the clinical and laboratory data can only be accessed by defined persons that have contributed to the project. These persons are two dedicated study nurses, the Principal Investigator and the Sponsor. Radiological data will be stored in the institutions’ PACS systems according to the institutional standard at the Balgrist University Hospital.

#### Case report forms

We will generate an electronic CRF in REDCap® for every participant and all data relevant to the study will be recorded by authorized persons. The participant ID numbers are automatically assigned in consecutive ascending order by the REDCap® system.

#### Analysis and archiving

For data analysis, we will export and analyze subject-related data from REDCap® in statistics software (IBM-SPSS and/or STATA). All health-related data will be archived in the REDCap® system for a minimum of 20 years. Before data export, we will remove all patient identifiers. Collection, disclosure and storage of data will be carried out in accordance with Swiss data protection regulations and the Human Research Act. The BioBank stores the intraoperative samples in accordance with laboratory guidelines as standard.

## Discussion

We will seek to demonstrate the noninferiority in the remission of infection of a shorter antibiotic treatment in adult patients with DFI, including DFO, with and without amputation [7], independently of surgical debridement, the level of arteriopathy and the causative pathogens. Importantly, all study participants will have professional and regular wound debridement, adequate off-loading, eventual revascularization, and a concomitant multidisciplinary surgical, re-educational, internist and infectiology surveillance. The studies will start at the Balgrist, but will be expandable to other settings with experience in DFI. Also, the secondary outcomes of adverse events and overall treatment costs will likely be less in the shorter antibiotic arms.

DFIs are associated with substantial morbidity, prolonged hospitalization, a life-long risk for lower extremity amputations and high financial costs [1, 8, 9]. When presented with a patient with a DFI, surgeons and physicians want to reduce the risk of poor outcomes. This often leads them to overprescribing antibiotic therapy [3]. This can take the form of prescribing an unnecessarily broad-spectrum regimen (often with combinations of agents), administering parenteral rather than oral therapy [10], or continuing therapy for a longer duration than necessary [1, 3, 8]. However, such overuse is not only ineffective, but is also associated with risks of adverse events, increased costs and promoting antibiotic resistance. Looking at the financial side, annual direct medical costs related to diabetes in the US alone were estimated at $176 billion in 2012 [11]. In a single hospital in Trinidad and Tobago, costs for the care of only 446 DFI patients was $14 million US dollars per year [12], which the authors extrapolated to represent 0.4% of the entire gross domestic product of that country. The direct antibiotic-related costs for a single DFI added up to $1000 Australian dollars in Australia [13].

In a recent prospective trial randomizing the use of topical gentamicin sponges (together with systemic antibiotics) for ulcerated DFIs, AEs occurred in 23% [14]. Looking at antibiotic-related AEs, studies have reported high rates of kidney injuries [15], selection of resistant pathogens such as methicillin-resistant *Staphylococci* or vancomycin-resistant *Enterococci* [16]. The incidence of resistant pathogens reached 15% and the rate of transient renal insufficiency reached 30% in one study [15]. Other author groups reported nausea, drug-induced hepatitis, *Clostridium difficile* colitis [17], and central line-related problems from intravenous therapy [3, 15] when treating orthopedic infections, including DFIs.

Current literature and expert opinions advocate 1–3 weeks of antibiotic therapy for soft tissue DFI and 4 to 6 weeks for bone infections, including toe arthritis [1–4, 8, 15]. The duration of the initial intravenous administration or an entire antibiotic course by oral antimicrobial agents alone had no effect on DFI recurrence [6, 10]. There seems to be no threshold for an optimal antibiotic duration, even when analyzing 1018 different DFI episodes in 482 patients [6]. In line with these findings, previously published studies in other fields of orthopedic infections equally failed to define an optimal duration of antibiotic therapy, such as in prosthetic joint [18] or fracture-device infections [19], septic bursitis [20], native joint septic arthritis [21], long bone osteomyelitis [22], or even open fractures [23]. All these infections are strongly associated with the presence of diabetes mellitus and its complications and thus require multidisciplinary management [24].

Likewise, when a less aggressive amputation is the goal, surgeons may face the problem that there is residual infection left, even if the amputation has been performed in apparently clean tissue or bone. Hence, in daily practice, the antibiotic prescription after toe amputation *in toto* ranges between some days of oral therapy to several weeks of intravenous administration. Moreover, the surgeons often ignore the ideal level of amputation to choose. Kowalski et al. demonstrated that patients with positive resection margins for residual postamputation osteomyelitis had more failures than those without (44% versus 15%, despite 2 weeks antibiotic therapy in both arms) [25]. Atway et al. reported a 41% incidence of positive bone margins among 27 transosseous amputations, compared to 23% following disarticulation [26]. Positive margins were associated with worse outcome despite 25 days of postsurgical antibiotic therapy. In contrast, Mijuskovic et al. showed that the assessment of residual bone infections might overestimate the risk of osteomyelitis as defined by histology because of contamination from soft tissue at the time of surgery [27]. According to a retrospective analysis of a Genevan database, antibiotics could be stopped immediately after amputation if the margins were clinically and visually clean [10, 28]. Clearly, the duration of antibiotic use after amputation for DFI osteomyelitis remains another unresolved issue.

Despite two prospective, randomized designs and 400 different episodes, we anticipate some limitations of our project. For example, patients who are treated outside of our center may have been lost to our follow-up. However, our center is the largest public hospital for DFI in the region, so this is unlikely to be a major bias. Additionally, our minimal follow-up time of 2 months is within the time window where most recurrences occur. Second, we will focus our study practically on moderate DFIs requiring referral to a tertiary center and potentially involving surgery. Thus, our data may not reflect outcomes related to mild DFI. Third, we decided against analyzing specific antibiotic agents or the role of specific pathogens. There is no evidence that any specific systemic antibiotic regimen is superior for DFI treatment, or for any specific pathogen [1, 16, 29, 30]. Fourth, pressure off-loading is crucial not only for prevention, but also for treating DFI. While the rationale of such measures is easily understandable, effectively implementing them depends on the patient’s adherence which we cannot monitor during the outpatient phase of the study.

In conclusion, we are confident that we can reveal clinically important answers to frequent questions regarding antibiotic use in DFIs, to ensure the quality of care, and to avoid unnecessary excesses in terms of examinations, microbiology, costs, surgery and antibiotic use.

## Data Availability

Minimal datasets will be available from the corresponding author upon reasonable request.
